# Therapeutic exercises in the clinical practice of Brazilian physical therapists in the management of rotator cuff tendinopathy: An online survey

**DOI:** 10.1371/journal.pone.0301326

**Published:** 2024-04-16

**Authors:** Denise Dal’Ava Augusto, Rodrigo Scattone Silva, Débora Pereira Pinheiro, Catarina de Oliveira Sousa

**Affiliations:** 1 Department of Physical Therapy, Federal University of Rio Grande do Norte, Natal, Rio Grande do Norte, Brazil; 2 Postgraduate Program in Physical Therapy, Federal University of Rio Grande do Norte, Natal, Rio Grande do Norte, Brazil; 3 Postgraduate Program in Rehabilitation Sciences, Health Sciences College of Trairi, Federal University of Rio Grande do Norte, Santa Cruz, Rio Grande do Norte, Brazil; Professionshøjskolen UCN: Professionshojskolen UCN, DENMARK

## Abstract

The objective of this study was to investigate how Brazilian physical therapists (PTs) use therapeutic exercises in the rehabilitation of individuals with rotator cuff (RC) tendinopathy. The study used an online survey with a mix of 62 open- and closed-ended questions divided into three sections: participant demographics, professional experience, and clinical practice in the rehabilitation of patients with RC tendinopathy. One hundred and fifty-nine Brazilian physical therapists completed the survey. Most of our sample recommended isometric exercises (69.9%) in the initial phase of rehabilitation and eccentric exercises (47.4%) in the advanced phase. However, there was a wide variability in determining the volume of exercises, particularly with isometric exercises. Most of our sample considered patient comfort and pain levels when adjusting exercise intensity, regardless of exercise type. The majority (48.40%) recommended weekly reassessment and modification of exercises. Additionally, despite pain being a key factor for discharge and the primary adverse effect of exercise, most of our sample would not discontinue exercises in case of pain during the early and late phases of rehabilitation. Despite the lack of consensus on some aspects, the clinical practice of our sample is in line with the current literature and practice in other countries. However, further research and implementation are crucial to enhance future rehabilitation outcomes, including exploring the exercise training volume, the safety and effectiveness of exercising with pain and identifying the optimal pain level for best results.

## Introduction

Shoulder pain is one of the most common musculoskeletal complaints, with a lifetime prevalence of up to 67% [1], and rotator cuff (RC) tendinopathy is among the most frequent diagnoses of shoulder pain. RC tendinopathy consists of tendinopathy of one or more of the four components of RC tendons, may or may not involve inflammation of the shoulder bursae, and can advance to partial or complete tendon rupture [2, 3]. In Brazil, a study conducted by Malavolta and colleagues [4] in patients with shoulder pain found a prevalence of 64.3% of RC related conditions, with 41.2% of cases being tendinopathy. Individuals with RC tendinopathy typically have reduced strength in shoulder muscles, functional limitation [5], lower quality of life [6] lower quality of sleep [7], and significant absenteeism from workplace [8]. These repercussions significantly affect individuals’ lives and impose a considerable burden on healthcare services [9]. Approximately 40% of those affected by RC tendinopathy experience pain and disability for more than 12 months, often requiring invasive interventions such as surgery or injections [10]. A potential factor in the development or persistence of pain is the presence of central motor alterations associated with RC tendinopathy which may contribute to the neuromuscular deficits associated with this disorder [3].

Recent systematic reviews have shown that progressive resistance exercises are effective for improving shoulder pain and function in patients with tendinopathy [11–14]. Passive techniques such as laser therapy, therapeutic ultrasound, shock-wave therapy, short waves, and microwaves have been shown to be ineffective for improving pain and function in patients with RC tendinopathy, which highlights the importance of active techniques such as resistance exercises for the management of this condition [15]. Despite being a primary intervention, there is great diversity regarding the type and parameters of resistance exercises [15, 16], both in isometric exercises [11] and in isotonic exercises [12, 17], with no consensus regarding the parameters.

Evidence-based practice entails decision-making based on clinical experience, patients’ preferences, and rigorous scientific research [18, 19]. In this context, it is important to investigate the approaches of physical therapists specializing in ​​musculoskeletal and/or orthopedic, traumatological, rheumatological, and sports rehabilitation. Specially, understanding how therapeutic exercises are employed in the rehabilitation of individuals with RC tendinopathy.

Online cross-sectional surveys were carried out with physical therapists from different locations around the world in order to identify their clinical approaches to managing pain and improving function in patients with RC tendinopathy [20–22]. Currently, Brazil has approximately 278,700 active physical therapists [23], however, it remains unknown whether Brazilian physical therapists are using progressive resistance exercises for the rehabilitation of patients with RC tendinopathy as well as which parameters are being used to guide its implementation. The objective of this study was to carry out an online survey to investigate if and how Brazilian physical therapists use therapeutic exercises in the rehabilitation of individuals with RC tendinopathy. This investigation will provide more detail about which criteria are being used for determining the initial load and progression of exercises, frequency, volume, identification of adverse effects and criteria for discharge, as well as recommendations of invasive interventions. To our knowledge, this is the first survey that investigates Brazilian physical therapists with this purpose. We hypothesized that, similar to other surveys [20–22], Brazilian physical therapists will use therapeutic exercise in their clinical practice, however, parameters of exercise will have a large variability since there is unclear scientific evidence in parameters for exercises in the rehabilitation of individuals with RC tendinopathy [14].

## Methods

### Study design

An open survey was constructed in accordance with the Checklist for Reporting Results of Internet E-Surveys (CHERRIES) [24] and the Strengthening the Reporting of Observational Studies in Epidemiology (STROBE) [25] recommendations in this cross-sectional study.

### Participants and survey design

Before submitting the research protocol to the research ethics committee, a pilot test was conducted with three Brazilian physical therapists who were PhD students and were not participating in the research group of the study. They completed the survey to identify potential errors or issues and to test for clarity and survey functionality. They spent between 10 and 15 minutes to complete the survey and the answers collected during this pilot phase were not included in the total study sample. Afterwards, the study was submitted and approved by the research ethics committee of the Federal University of Rio Grande do Norte (protocol CAAE—50945421.2.0000.5537 and approval no. 4.987.424).

A convenience sampling of Brazilian physical therapists who worked in ​​musculoskeletal and/or orthopedic, traumatological, rheumatological, and sports rehabilitation were considered eligible for this study. Those who reported not treating at least one patient with shoulder pain per month and those who filled at least three questions of the survey with incomplete answers were excluded.

The survey was developed in Brazilian Portuguese and disseminated through social networks and emails sent to physical therapists affiliated to the Brazilian Federal Council of Physical Therapy and Occupational (COFFITO-Brazil) and Brazilian National Society of Sport Physical Therapy (SONAFE-Brazil). The physical therapists received an email with the general objectives of the study and the eligibility criteria and the survey link. Those who agreed to participate in the study read and signed the informed consent form providing their consent prior to participation. Participants were informed of the objectives, risks, and benefits of the study and had their confidentiality protected. Participants did not receive any compensation (payment or non-monetary reward) for their participation. The data generated by the answers were password-protected, and only the first author and the corresponding author of the paper had access. The survey was hosted by Google Forms platform online between September 22, 2021 and April 3rd, 2022. Online surveys offer an efficient option for remote data collection and increase accessibility for some populations [26].

The survey was created by three physical therapists with 13–15 years of clinical experience in the field and based on current recommendations for exercise-based treatment of RC tendinopathy [11, 13, 16, 27, 28]. The questions were also based on those of similar online surveys recently conducted in other countries [21, 22]. The survey (S1 Table) comprised a total of 62 questions, blending open-ended and closed-ended formats. All main questions were mandatory to prevent incomplete responses and participants were able to review and change their answers using a back button. The form had a total of 24 pages, with an average of 5 questions per page, organized into three sections: 1) demographic characteristics, 2) professional experience, and 3) clinical practice, based on a clinical case. Briefly, the form included questions about physical therapists’ clinical experience with regards to their recommendations of resistance exercises for rehabilitation, i.e., types of exercise and their application in the different phases of rehabilitation, parameters of frequency and volume of training, the criteria for physical therapy discharge, the adverse effects found for exercise therapy, among others (S1 Table).

### Data analysis

All individual answers were exported to Excel. The duplication of responses was controlled through participants’ access email. Limitations in our online tool prevented us from recording unique visitor numbers; thus, we could not calculate view, participation, and completion rates. Data were analyzed descriptively, and the responses are presented with absolute and relative frequencies. For the close-ended questions, the proportion of respondents (%) selecting each response option was calculated. For the open-ended questions, we used a content analysis, in which the different responses were grouped; thus, the same/similar answers were identified and coded into categories. The proportion of respondents (%) providing each category of responses was then calculated [21].

## Results

### Characterization of participants and their professional experience

One hundred and seventy-one physical therapists participated in the survey. Of these, five were excluded for reporting that they did not work in ​​musculoskeletal and/or orthopedic, traumatological, rheumatological, and sports rehabilitation; and seven were excluded for reporting that they did not treat patients with shoulder pain. No participant was excluded due to incomplete answers.

Therefore, a total of 159 questionnaires were analyzed. The mean age of the participants was 35 years-old, varying between 22 and 68 years, with the majority being women (52.83%) and living in cities in the Northeast region of Brazil (Table 1).

**Table 1:** Individual, demographic and professional characteristics of the sample.

Variables	Mean (SD) / Frequency–n (%)
**Individual and demographic characteristics**	**n = 159**
**Age (years)**	35 (7)
**Gender**	
Male	75 (47%)
Female	84 (53%)
Other	—
**Region**	
Northeast	80 (50%)
North	3 (2%)
Southeast	57 (37%)
South	12 (7%)
Central-West	7 (4%)
**Professional experience**	
**Level of Education**	**n = 159**
Bachelor’s Degree	40 (25%)
Postgraduate Specialization	79 (50%)
Orthopedics and Traumatology	40 (51%)
Sports	9 (11%)
Rheumatology	1 (1%)
Others	29 (37%)
Master´s Degree	23 (14%)
Orthopedics and Traumatology	9
Sports	5
Rheumatology	3
Others	6
PhD	15 (9%)
Orthopedics and Traumatology	4
Sports	3
Rheumatology	3
Others	5
Post Doctorate	2 (1%)
**Time working as physical therapist**	**n = 159**
Less than 1 year	10 (6%)
Between 1 and 5 years	45 (28%)
Between 5 and 10 years	26 (16%)
Between 10 and 15 years	38 (24%)
More than 15 years	40 (25%)
**Time working in musculoskeletal rehabilitation field**	**n = 159**
Less than 1 year	13 (8%)
Between 1 and 5 years	48 (30%)
Between 5 and 10 years	29 (18%)
Between 10 and 15 years	32 (20%)
More than 15 years	37 (23%)
**Special interest in shoulder rehabilitation**	**n = 159**
Yes	121 (76%)
No	38 (24%)
Type of healthcare service*	
Public	50 (29%)
Private	153 (90%)
**Place where professional activity is carried out** *	
Clinic	130 (50%)
In-home patient care	90 (35%)
Hospital	21 (8%)
Tele rehabilitation	5 (2%)
Other**	14 (5%)
**Mean of patients with shoulder pain seen per month**	**n = 159**
Up to 5	86 (54%)
From 6 to 10	46 (29%)
From 11 to 15	17 (11%)
More than 15	10 (6%)

* Participants were permitted to provide multiple answers.

** Other places mentioned: Office, university clinic, club, gym, crossfit box and pilates studio.

Regarding professional experience, approximately 65% had more than 5 years of experience as physical therapist and approximately 50% had a specialist degree, with the majority (50.63%) being in the area of ​​orthopedics and traumatology. Most of the physical therapists (76.10%) reported a special interest in shoulder rehabilitation, treating, on average, up to 5 patients (54.09%) with shoulder pain per month.

### Clinical practice based on clinical case

#### Indication of exercises according to the phases of the rehabilitation

**Fig 1:**
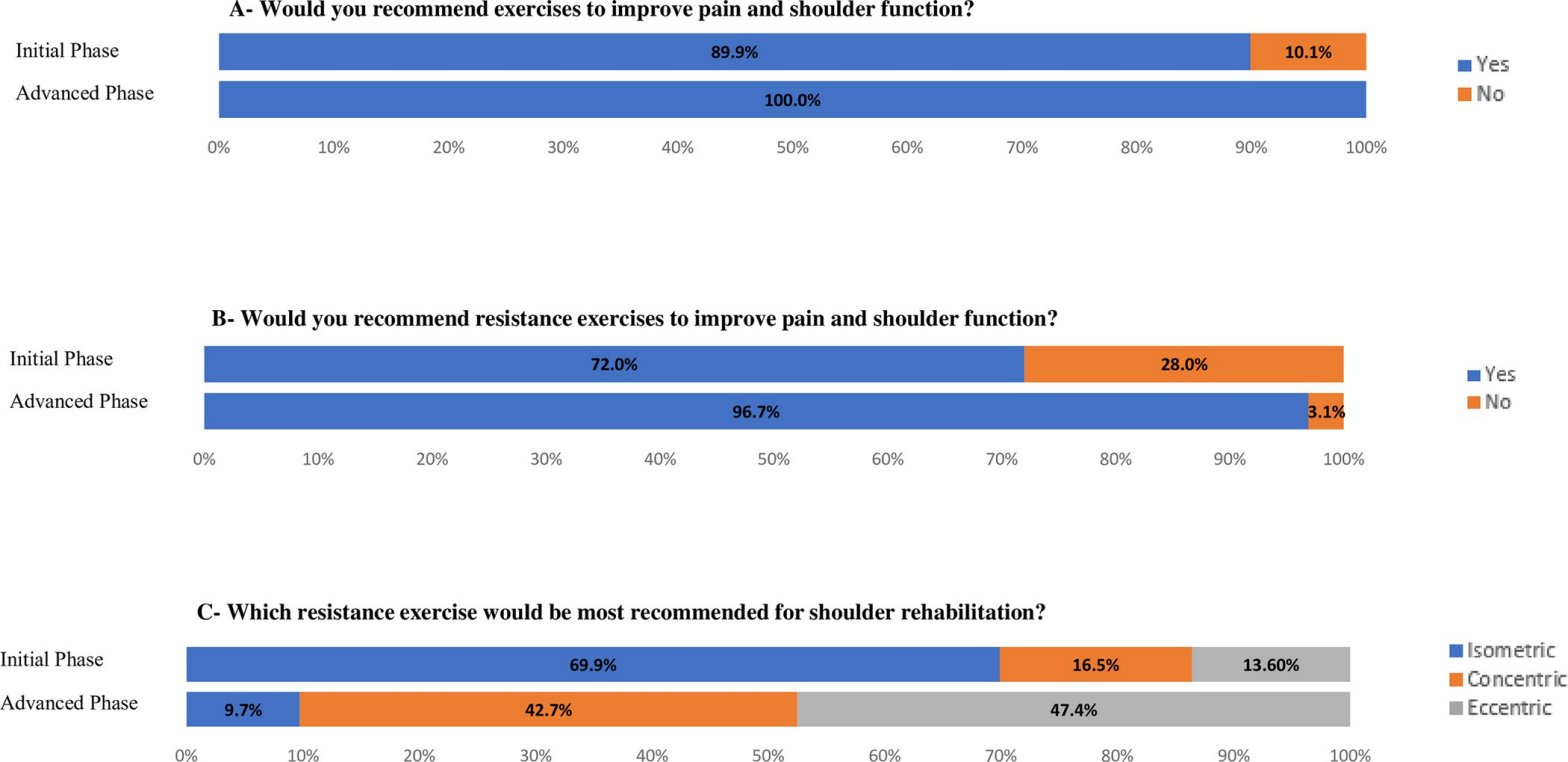
Indication of exercises according to the phases of rehabilitation.

As described in Fig 1, one hundred and forty-three physical therapists (89.9%) would recommend exercises in the initial phase of rehabilitation for individuals with RC tendinopathy. Among these, 72% would recommend resisted RC exercises, with the majority (69.9%) recommending isometric, followed by concentric (16.5%) and eccentric (13.6%) exercises, respectively. In the advanced phase, all 159 physical therapists (100%) would recommend the use of exercises and the vast majority (96.7%) would recommend resisted RC exercises, with the majority (47.4%) recommending eccentric exercises, followed by concentric (42.7%) and isometric (9.7%), respectively.

#### Criteria for carrying out the exercises

**Fig 2:**
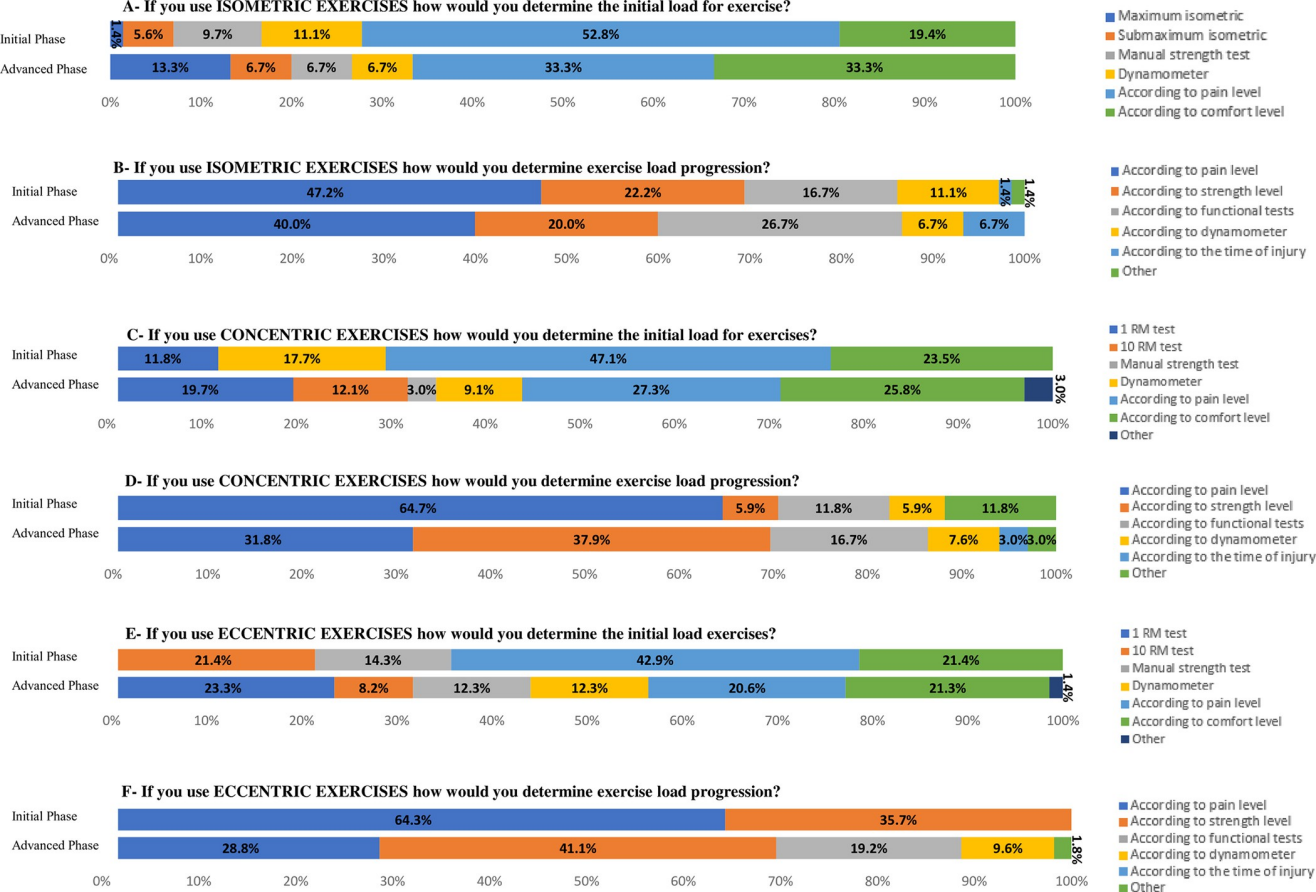
Criteria for determining the initial load and progressing exercise load according to each type of exercise and phase of rehabilitation.

Out of the 72 physical therapists who would recommend isometric exercises (Fig 2), in the initial phase of rehabilitation, the majority considers the patient’s pain level to determine the initial exercise load (52.8%) and load progression throughout the treatment (47.2%). As for the advanced phase of rehabilitation, out of the 15 physical therapists who would recommend isometric exercises, five (33.3%) consider the patients’ pain level and five (33.3%) consider the level of comfort to determine the initial exercise load. For load progression, the majority (40%) reported they consider patient’s pain level as the main criterion.

Of the 17 physical therapists who would recommend concentric exercises (Fig 2), in the initial phase of rehabilitation, the majority would also use patient’s pain level for initial determination of load (41.7%) and for its progression (64.7%). In the advanced phase of rehabilitation, among the 66 physical therapists who recommend concentric exercises, the majority (27.3%) would determine the initial load of the exercises based on patient’s pain level, followed by the level of comfort (25.8%). As for the progression of exercise load, the majority of physical therapists (37.9%) would consider the level of strength, followed by the level of pain (31.8%).

Regarding the 14 physical therapists who would recommend eccentric exercises (Fig 2), in the initial phase of rehabilitation, the majority considered the patient’s pain level to determine the initial exercise load (42.9%) and its progression (64.3%). In the advanced phase of rehabilitation, among the 73 physical therapists who would recommend eccentric exercises, the responses were substantially diverse to determine the initial exercise load. The progression of the load would be determined by the majority (41.1%) based on the strength level followed by the level of pain (28.8%).

**Fig 3:**
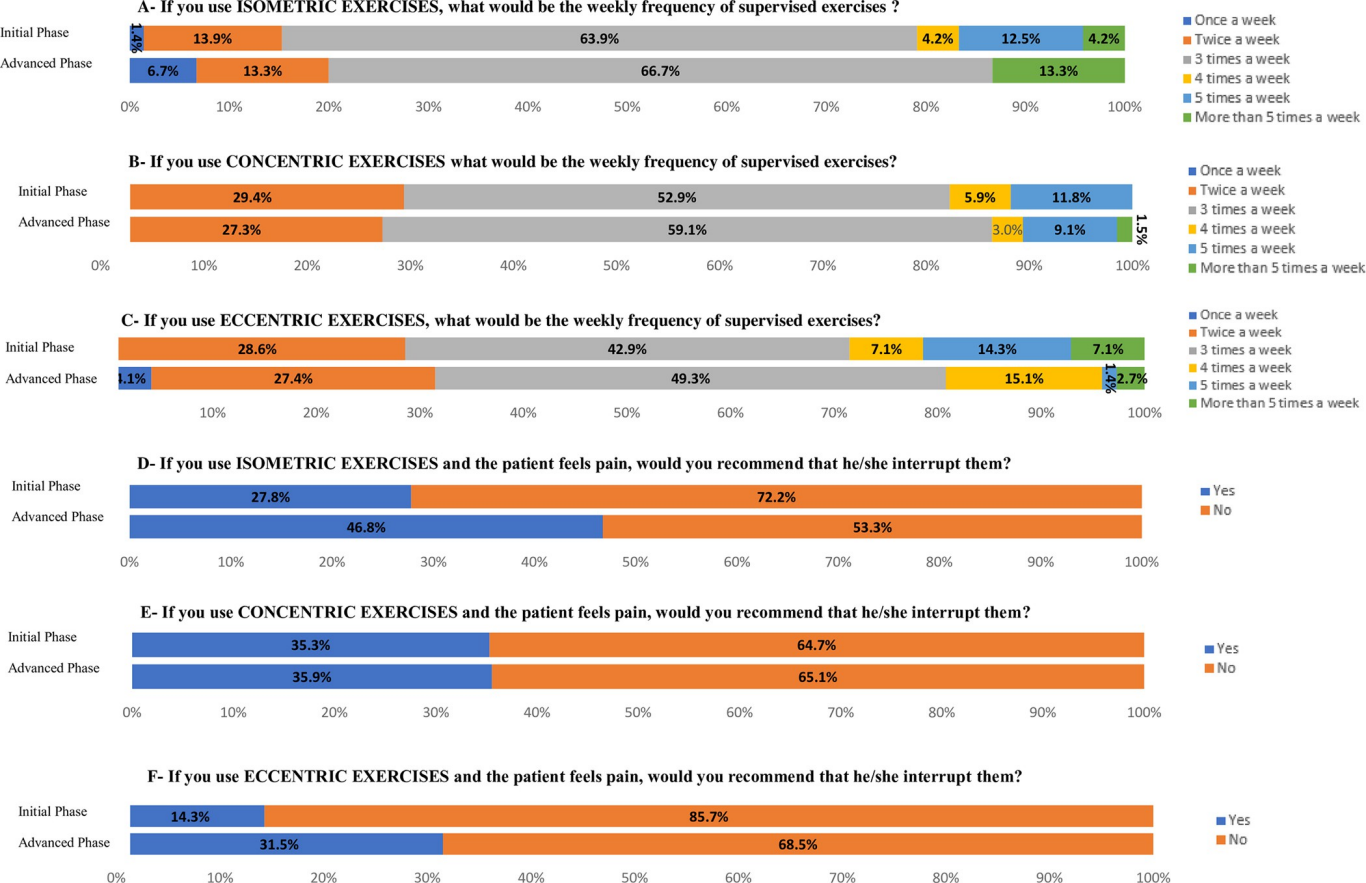
Weekly frequency of exercises and recommendation for interrupting exercises in the presence of pain, according to each type of exercise and phase of rehabilitation.

Regardless of exercise type, the majority of physical therapists would recommend exercises 3 times a week and would not interrupt resistance exercises in the presence of pain, both in the initial and advanced phases of rehabilitation (Fig 3).

As for the training volume for RC resistance exercises, the answers were substantially diverse.

**Fig 4:**
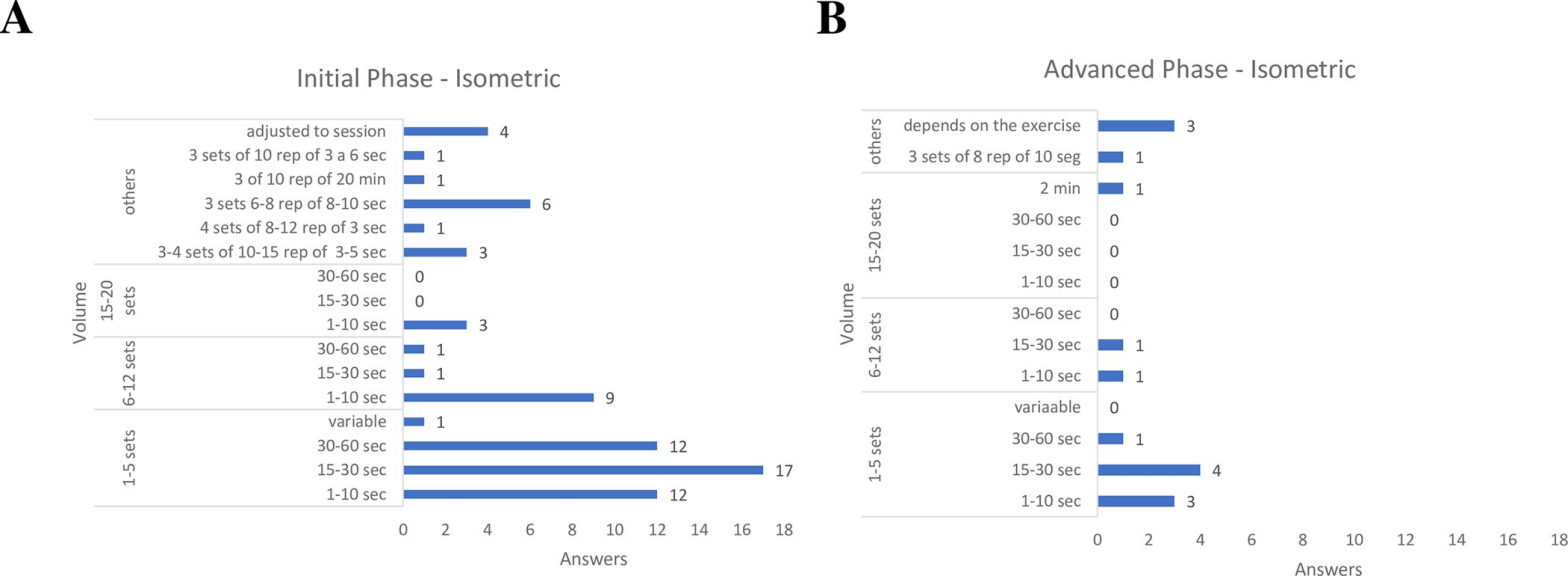
Training volume for isometric exercise, both for early and advanced phases of rehabilitation.

Among the physical therapists who would indicate isometric exercises (Fig 4) in the initial and advanced phases of rehabilitation, the majority recommended between 1 and 5 sets with 15 to 30 seconds of contraction.

**Fig 5:**
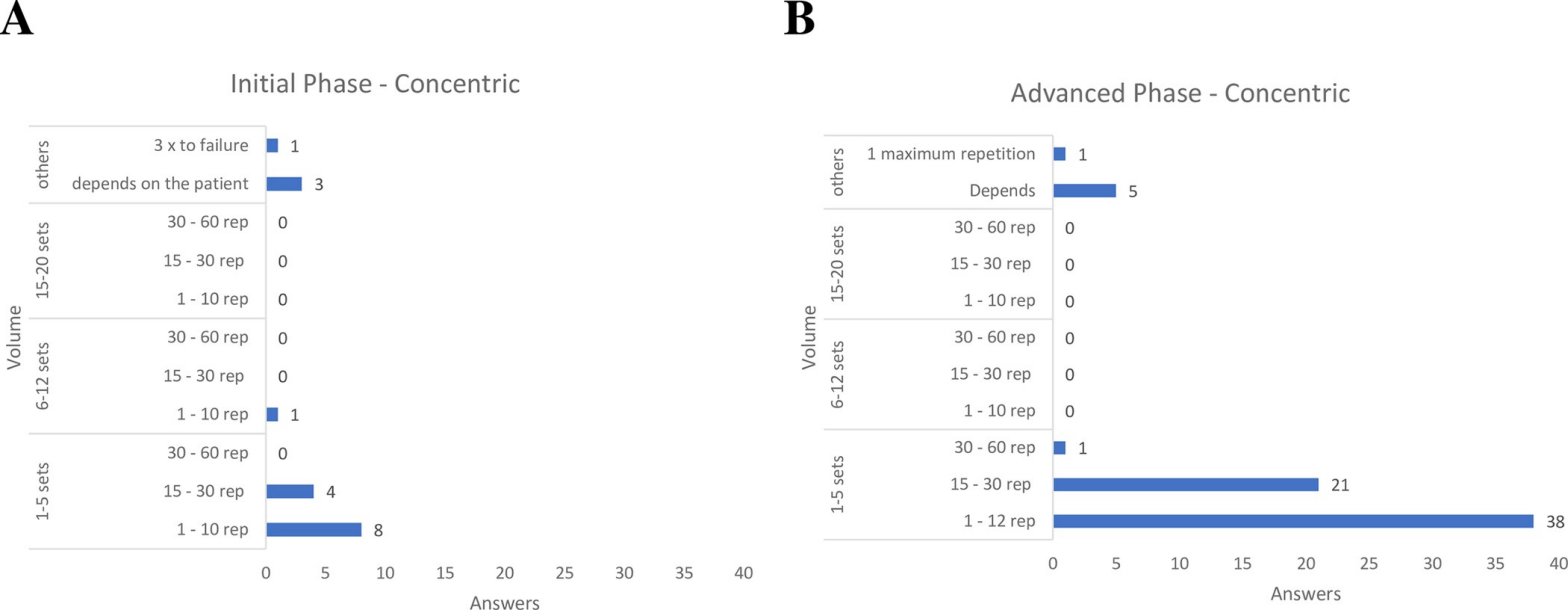
Training volume for concentric exercise, both for early and advanced phases of rehabilitation.

For concentric exercises (Fig 5), in both phases of rehabilitation, most physical therapists recommended from 1 to 5 series with 1 to 10 repetitions for initial phase and 1 to 12 repetitions for advanced phase.

**Fig 6:**
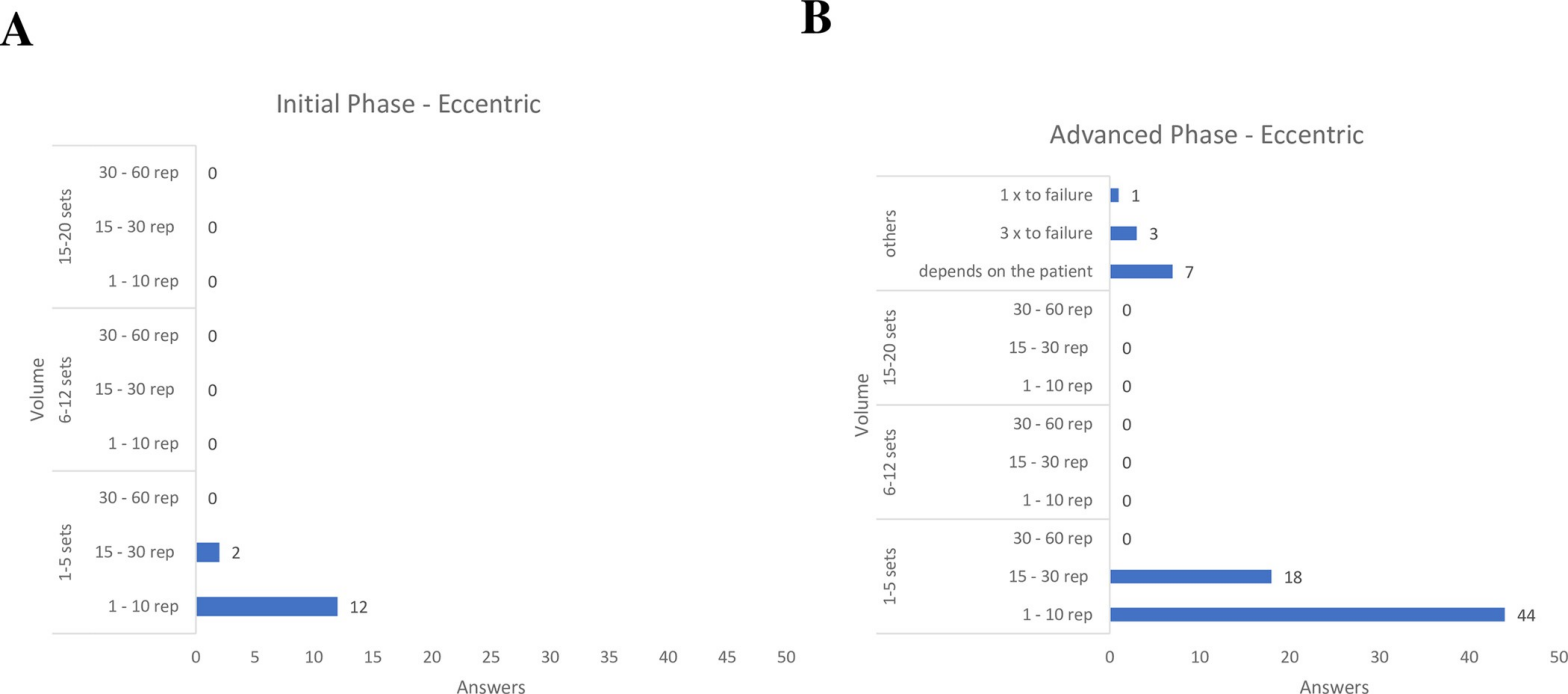
Training volume for eccentric exercise, both for early and advanced phases of rehabilitation.

For eccentric exercises (Fig 6), in both phases of rehabilitation, the majority of physical therapists recommended between 1 and 5 sets, with repetitions between 1 and 10.

#### Additional exercises and therapeutics

**Fig 7:**
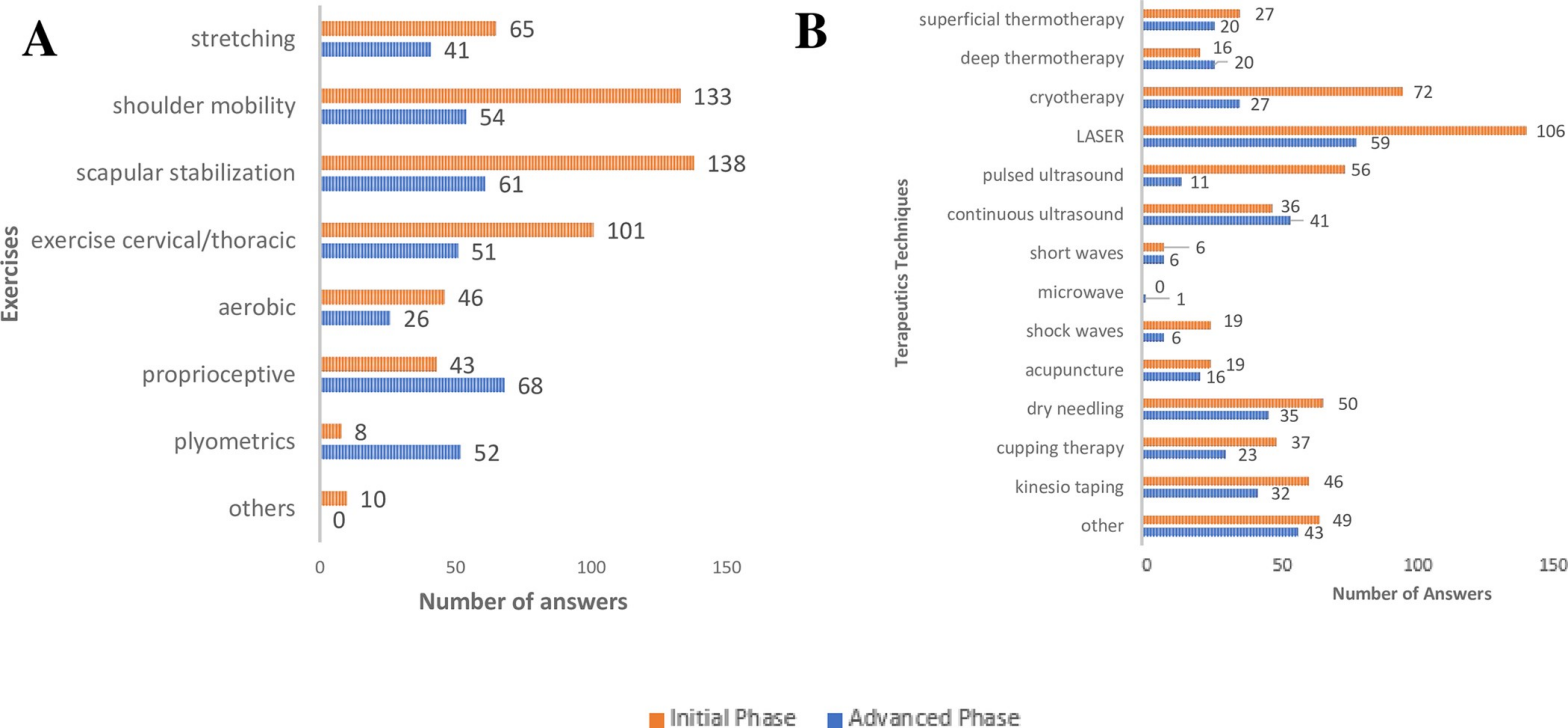
Other exercises and therapeutic techniques recommended by Brazilian physical therapists for the treatment RC tendinopathy, both in the initial and advanced phases of treatment.

In addition to resistance exercises for the RC, other exercises would also be recommended both in initial and advanced phases of rehabilitation (Fig 7), being the most mentioned scapula stabilization (25.37%—initial and 17.28%—advanced), shoulder mobility (24.45%—initial and 15.30%—advanced) and cervical and/or thoracic spine exercises (18.57%—initial and 14.45%—advanced). Also, other techniques would also be recommended (Fig 7), both in the initial and advanced phases, such as laser (19.67%—initial and 17.35%—advanced), cryotherapy (13.36%—initial and 7.94%—advanced) and pulsed ultrasound (10.39%—initial and 3.24%—advanced), among others.

For home care, 2.4% (n = 10) of the physical therapists would recommend rest, 12.5% ​​(n = 52) stretching exercises, 13.70% (n = 57) 1 to 2 RC strengthening exercises, 21.39% (n = 89) 3 to 5 RC strengthening exercises, 27.16% (n = 113) education and modification regarding the way of sleeping and 22.85% (n = 95) modification of movements and activities of daily living. It was possible to provide more than one recommendation. Thus, 24.53% of our sample would suggest 2 interventions for patients to perform at home, whereas 56.60% would recommend 3 or more home interventions.

**Fig 8:**
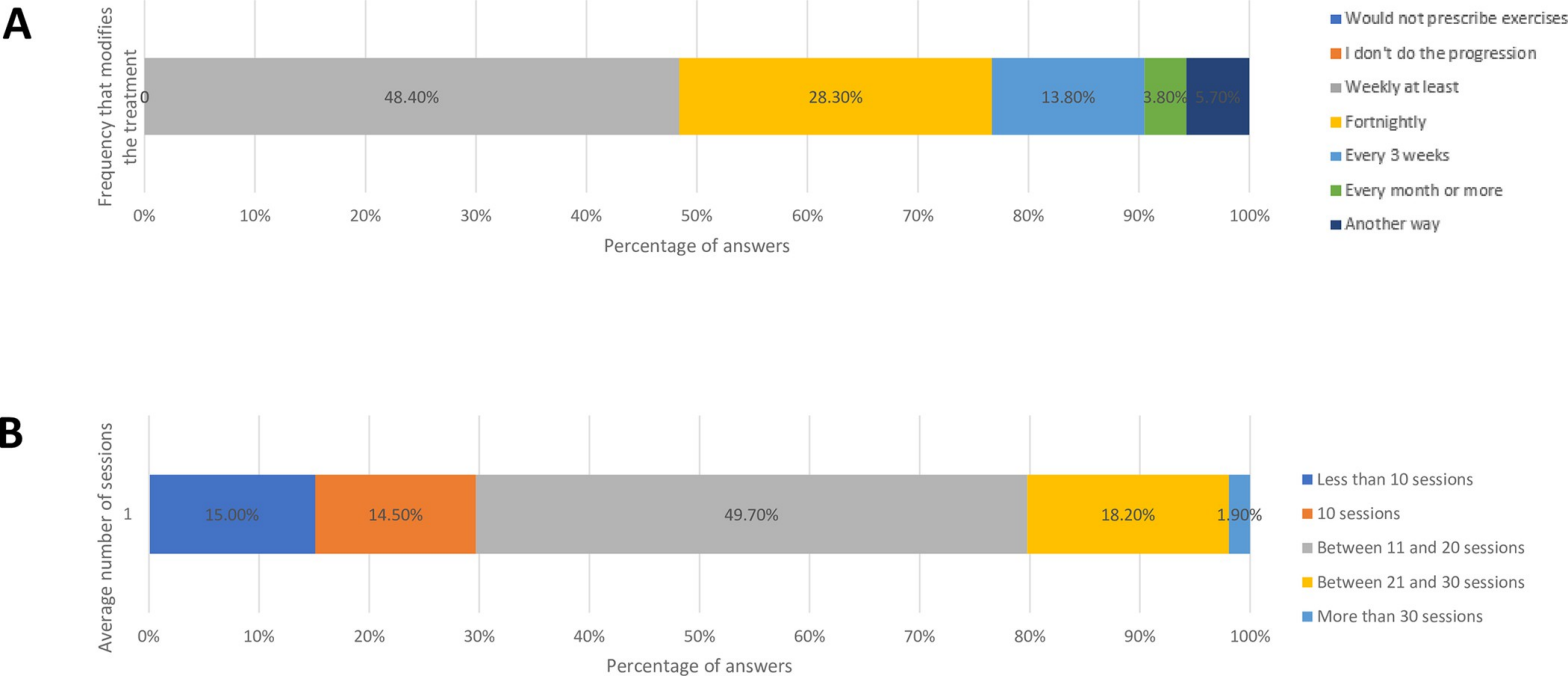
Frequency for assessing and adjusting exercises and mean number of sessions needed to improve pain and function in the treatment of RC tendinopathy.

As for the assessment and modification of exercises during the rehabilitation (Fig 8), the majority of physical therapists (48.40%) reported that they reassess patients on a weekly basis and that it is necessary to reassess between 11 and 20 sessions to improve patient’s pain and function (49.70%).

#### Adverse effects of exercises and criteria for discharge

**Fig 9:**
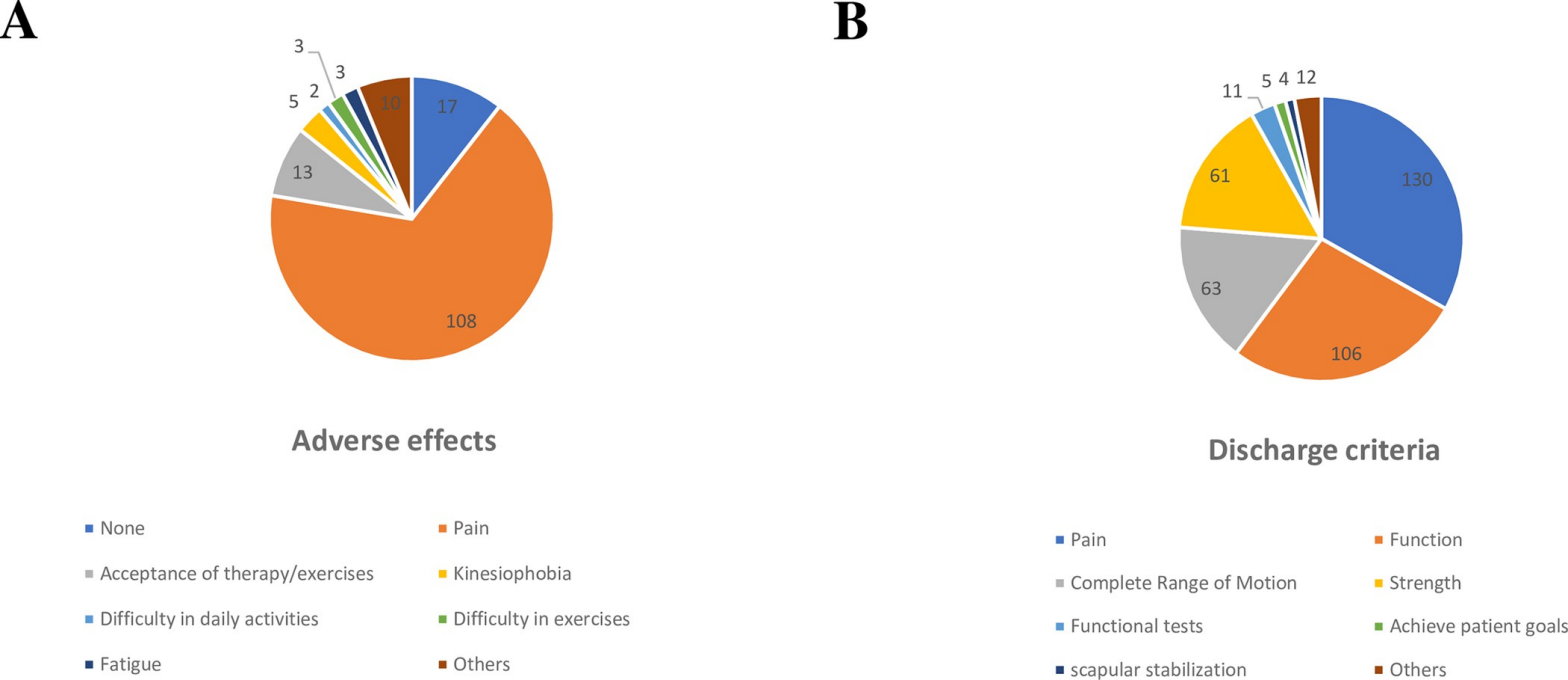
Adverse effects found with the use of the exercises and discharge criteria for patients with RC tendinopathy.

Among possible adverse effects that physical therapists encounter from exercises for RC tendinopathy, pain was the most frequent (Fig 9). Regarding discharge criteria, the primary consideration were pain reduction and improvement in function (Fig 9).

#### Recommendation for invasive interventions

Of the 159 physical therapists, 94.97% (n = 151) answered that they would not indicate invasive interventions during conservative treatment. However, when conservative treatment fails, 58.49% of the physical therapists (n = 93) would recommend invasive interventions, where the majority (n = 89) would refer the patients to a physician.

## Discussion

Our sample of Brazilian physical therapists widely use therapeutic exercises in their clinical practice to treat patients with RC tendinopathy, as do physical therapists in other countries [20–22]. In addition, the recommendations of our sample are in accordance with those presented in studies that highlight the importance of exercises in the rehabilitation of individuals with RC tendinopathy [13, 16, 27–29].

Although more than 75% of the physical therapists reported a special interest in treating patients with shoulder pain, there was great variability among responses regarding types and dosage of exercises used, which also reflects the lack of consensus in the literature about the best exercises and the best parameters to treat patients with RC tendinopathy [13, 28, 30]. Studies using concentric/eccentric exercises often lack specified loads, employing elastic bands, hindering standardization [31, 32]. Other studies using repetition maximum lack consensus on progression [17, 33]. Studies with isometric exercise also lack load consensus, with some using 50–75% of maximal strength [34] and others omitting load details [35, 36].

Similar to the UK clinical practice [21] as well as Belgians and Dutch [20] physical therapists, our sample of Brazilian physical therapists take an average of 11 to 20 sessions to improve patients’ pain and function.

### Indication of the exercises according to the phases of the rehabilitation

Most physical therapists in our sample recommended isometric exercises in the initial phase of rehabilitation, and eccentric exercises in the advanced phase. Isometric exercises have been widely used in different types of tendinopathies, such as Achilles and patellar tendons [37, 38] and also in RC [36], mostly due to its analgesic effects [39, 40]. Possibly due to the lack of joint movement involved, the physical therapists incorporated isometric exercises as part of the initial phase of rehabilitation. In the advanced phase of rehabilitation, the choice of eccentric exercises may reflect the possibility of applying greater load to the tendon, which could explain and favor a reduction in pain due to greater strengthening of the tendon [41]. Eccentric exercises were also widely used for various types of tendinopathies, but the literature is not clear regarding their superior effects compared to other types of exercises [42–44]. Regardless, eccentric exercises were the most cited among Australian physical therapists in the treatment of individuals with RC tendinopathy, independent of the treatment phase [22].

Other types of exercises were also recommended in our survey, with scapular stabilization and shoulder mobility exercises being the most frequently cited in both the initial and advanced phases. Recently, interest in scapular stabilization exercises has grown in the literature, and although there is conflicting evidence of their effectiveness [45–48], they have been widely applied [20, 21].

According to a meta-analysis conducted by Desjardins-Charbonneau et al. [49], manual therapy, which involves shoulder mobilization among other techniques, was effective in relieving pain in patients with shoulder tendinopathy. Furthermore, shoulder joint mobilization also improved shoulder function and reduced pain, in patients with shoulder pain [50]. The passive movements produced by manual techniques result in pain reduction through the activation of mechanoreceptors that inhibit the painful stimulus through the pain gate mechanism, in addition to also improving joint nutrition through the mobilization of synovial fluid [50]. However, the shoulder mobilization technique becomes more effective when associated with other techniques such as supervised exercises, for example [49, 50].

Regarding the use of other techniques associated with exercise therapy, the main recommendations in our sample were laser, cryotherapy, pulsed ultrasound and dry needling in both phases of rehabilitation. According to Bury and Littlewood [21] there was a clear decline in the use of other therapeutic modalities, compared to another study conducted by the same group [13], with the most notable decline for acupuncture, electrotherapy, corticoid injection, mobilization, and massage. This finding reflects the current evidence regarding the lack of clinical efficacy of passive therapeutic modalities [51–53], however these techniques were still frequently cited by our sample of Brazilian physical therapists.

As for home exercise program for patients, our sample recommended education and modifications related to sleeping position as well as adjustments of movements and activities of daily living, which corroborates with some clinical guidelines [29, 54, 55] and with the therapeutic approach in other countries [20–22]. According to Hopman and colleagues [54] continuing activities that worsen symptoms can be a contributing factor to the failure of conservative treatment, thereby justifying the need to modify habits.

### Criteria for carrying out the exercises

There was a high variability about the parameters for exercises, mainly with regards to the training volume for isometric exercises. Most physical therapists in our sample recommended isometric exercises in 1 to 5 sets of 15 to 30 seconds of contraction, both in the initial and advanced phases of rehabilitation; however, many other responses were reported. This variability was also verified in other surveys carried out in other countries [20–22], and also in clinical studies [28, 30].

Regardless of the number of sets, load, and duration of isometric contraction, a reduction in pain in response to this type of exercise has been observed in different regions of the body. Experimental pain was improved by performing isometric gripping exercises for 2 sets of 2 minutes [56]; isometric exercise with contraction at 21% of the maximum isometric force applied until exhaustion (maximum of 5 minutes) increased the pain threshold on the quadriceps femoris [57]; and performing isometric exercise for more than 3 minutes resulted in hypoalgesic effects on quadriceps pain [37]. When compared with isotonic exercises in individuals with lower limbs tendinopathy, isometric exercises presented greater hypoalgesic effect [37, 58]. Although isometric exercises have been used in the treatment of RC tendinopathy, studies on this topic are scarce in the literature [36].

Regarding concentric and eccentric exercises, most Brazilian physical therapists in our sample recommended 1 to 5 sets with 1 to 10 repetitions, both in the initial and advanced phases of rehabilitation, similar to the current literature on RC tendinopathy [31, 32] and shoulder impingement syndrome [41, 59–61] previous studies have utilized concentric and eccentric exercises employing 3 sets of 10 repetitions. However, a lack of consensus in the literature regarding the optimal training volume using concentric and eccentric exercises for patients with RC tendinopathy is still evident [41].

Patients’ pain and comfort were used as an exercise prescription parameter, both for determining the exercise type and for load progression, in isometric, concentric and eccentric exercises. This is in line with the findings of Smythe et al., [22] who observed similar results in the initial and advanced phases of treatment of RC tendinopathy patients. According to Lewis [3], RC tendinopathy can present several clinical phases with different stages of symptoms; thus, in more symptomatic phases, pain can be a key point in determining exercise parameters and in less symptomatic phases, functional capacity can be used more.

Another point cited for load progression, especially in concentric and eccentric exercises, was muscle strength, which can be justified by the rapid adaptation to resistance training, which requires changes in the variables of volume and intensity to ensure training progression [62]. Further research is needed to understand the main criteria that should be used to guide exercise prescription for people with different clinical stages of RC tendinopathy.

An important result of this study was that most physical therapists in our sample would not interrupt the exercises in the presence of pain during their execution. A recent systematic review evaluating the effect of exercise with and without pain concluded that exercise with pain results in better short-term outcomes and equivalent long-term outcomes [63]. Also, clinical shoulder experts recommend mild to moderate pain (less than 4/10 on a numerical pain scale) during exercises, and the pain should decrease to baseline levels within 12 hours [64]. However, a study recommends that pain should not be allowed during shoulder exercise [65]. Thus, studies should be conducted to assess whether exercise with pain is secure and leads to superior outcomes, as well the optimal level of pain during exercise.

Our sample of Brazilian physical therapists recommend reassessing and modifying the exercises every week, which corroborates the current literature and the principle of gradually increasing the load [29, 54].

### Adverse events of exercise and criteria for discharge

Most physical therapists in our sample report pain as the main adverse effect of exercise during the rehabilitation of patients with RC tendinopathy, which is in contrast to recent systematic reviews [12, 17] that found no adverse events as a consequence of resistance exercises.

As for the discharge criteria, most in our sample take into account the improvement in pain and function, which has been the main outcome variable in several studies that use exercises [11, 34, 36]. However, pain and function assessment involves diverse methods and tools, making it challenging to definitively determine the criteria applied within our sample.

### Recommendations for invasive interventions

Similar to Australian physical therapists, our sample does not advocate for invasive techniques during conservative treatment [22]. However, some studies have recommended the application of corticoid injections as part of the initial treatment [65], while others only recommend it after no improvement with conservative treatment [54].

## Limitations

A limitation of this survey is the fact that the majority of participants were from the Northeast region of Brazil, which may not represent the clinical practice of professionals who work with shoulder rehabilitation in other parts of Brazil. Furthermore, we couldn’t determine the response rate among Brazilian physical therapists in musculoskeletal, orthopedic, traumatological, rheumatological, and sports rehabilitation fields. Also, 76% of our sample exhibited a particular interest in treating patients with shoulder pain, which may have introduced a selection bias. This interest may have resulted in these professionals being more updated with the most current recommendations in the literature.

We acknowledge that we did not explicitly state in the survey that we considered initial and advanced phases of rehabilitation based on stages of irritability described in McClure and Michener [66]. So, each physical therapist may have applied their own set of criteria for phases of rehabilitation, which could have influenced the results.

Despite this, this study is a first step towards a better understanding of how Brazilian physical therapists treat patients with RC tendinopathies in comparison to the best reviews and consensus in the field.

## Conclusion

Most Brazilian physical therapists from our sample incorporate RC strengthening exercises in their rehabilitation programs, which is consistent with current literature recommendations. Isometric exercises are typically recommended for the initial phase, while eccentric exercises are recommended for the later phases. The parameters used for these exercises vary significantly in relation to the existing literature. Our study found that while patient pain and comfort guide exercise load progression, they do not consistently dictate when to interrupt exercises during execution. The discharge criteria most often consider patients’ improvement in both pain and function. Despite the lack of consensus on some aspects, our findings indicate that clinical practice of Brazilian physical therapists is in line with the current literature and practice in other countries. However, further research and implementation are crucial to enhance future rehabilitation outcomes, including exploring the exercise training volume and the safety and effectiveness of exercising with pain and identifying the optimal pain level for best results.

## Supporting information

S1 Table Questionnaire on clinical practice during the rehabilitation of patients with RC tendinopathy applied to Brazilian physical therapists. (DOCX)

S1 Checklist Checklist for Reporting Results of Internet E-Surveys (CHERRIES). (DOCX)

S2 Checklist STROBE statement—Checklist of items that should be included in reports of observational studies. (DOCX)

S1 Fig The survey announcement. (TIF)
