# Supplementary material for: Therapeutic exercises in the clinical practice of Brazilian physical therapists in the management of rotator cuff tendinopathy: An online survey
Source: PLoS One. 2024 Apr 16;19(4):e0301326. doi: 10.1371/journal.pone.0301326 (PMC11020769; doi:10.1371/journal.pone.0301326)
Supplement: S1 Table — (DOCX) [file pone.0301326.s001.docx]

**Table S1:** **Questionnaire on clinical practice during the rehabilitation of patients with RC tendinopathy applied to Brazilian physical therapists.**

| **Individual and demographic characteristics** | **Answer options** | |
| --- | --- | --- |
| 01- How old are you? | **---** | |
| 02- What is your gender? | Masculine  Feminine  Other | |
| 03- Where do you live? City and State. | **---** | |
|  |  | |
| **Professional experience** |  | |
| 04- Do you work in the field of musculoskeletal rehabilitation? | Yes  No | |
| 05- How many patients with shoulder pain, on average, do you see per month? | Up to 05  06 – 10  11 – 15  More than 15  I don't see patients with shoulder complaints | |
| 06- What is your highest level of educational attainment? | Bachelor's Degree  Postgraduate Specialization  Master Degree  PhD  Other | |
| 07- What is the total number of hours for the specialization? | ≥ 360 h  < 360 h | |
| 08- What is your area of ​​specialization/master's/PhD? | Orthopedics and Traumatology  Sports  Rheumatology  Other | |
| 09- How long have you been working as a physiotherapist? | > 1 year  1 – 5 years  5 – 10 years  10 – 15 years  < 15 years | |
| 10- How long have you been working in the musculoskeletal rehabilitation field? | > 1 year  1 – 5 years  5 – 10 years  10 – 15 years  < 15 years  I do not work in this field | |
| 11- Do you have a special interest in shoulder ​rehabilitation? | Yes  No | |
| 12- What type of healthcare service do you develop your professional activity? | Public  Private | |
| 13- Place where professional activity is carried out? | Clinic  In-home patient care  Hospital  Telerehabilitation  Other | |
|  |  | |
| **Clinical practice – Based on the clinical case**  “A 52-year-old male patient, with a medical diagnosis of RC tendinopathy and magnetic resonance imaging compatible with supraspinatus and infraspinatus tendinopathy, attended physical therapy with a history of insidious onset pain in the right shoulder 4 months before , in the anterolateral region of the shoulder without complaints of irradiation. Patient presents pain with active movement of the right shoulder, mainly in activities that require movements above the head, with the hand on the back or in activities to reach an object. He also mentions pain when sleeping on the affected side. It has a full active range of motion, but with a painful arc between 60 and 120º of arm elevation and maintained passive range of motion. Cervical RoM is complete and pain free. With regard to muscle strength, he has weakness for abduction and lateral rotation movements, with strength grade 4 on the Daniels scale (4/5).” |  |  |
| 14- What would you recommend as home care for this patient? | Rest  stretching exercise  From 1 to 2 rotator cuff strengthening exercises  From 3 to 5 rotator cuff strengthening exercises  Education and modifications of way of sleep  Modification of gestures and activities of daily living |  |
| 15- Would you recommend exercises in the initial phase of rehabilitation to improve shoulder pain and function? | Yes  No |  |
| 16- Would you recommend resistance exercises for the rotator cuff in the initial phase of rehabilitation to improve shoulder pain and function? | Yes  No |  |
| 17- Which resistance exercise would be most recommended for rehabilitation of shoulder tendinopathy, in the initial or phase? | Isometric  Concentric  Eccentric |  |
| 18- If you use resistance exercises **isometric**, in the initial phase of rehabilitation, and the patient feels pain during the exercises, would you recommend that he/she interrupt them? | Yes  No |  |
| 19- If **isometric** exercises are utilized during the initial phase of rehabilitation, how would you determine the initial load of the exercises? | Maximum isometric  Submaximum isometric  Manual Muscle Testing  Dynamometry assessment  According to the level of pain  According to the level of strength  Other |  |
| 20- If **isometric** exercises are utilized during the initial phase of rehabilitation, how would you determine exercise load progression? | According to the level of pain  According to the level of strength  According to the functional tests  According to the dynamometry assessment  According to the time of injury  Other |  |
| 21- If **isometric** exercises are utilized during the initial phase of rehabilitation, what would be the appropriate number of sets and duration of each isometric exercise? | --- |  |
| 22- If **isometric** exercises are utilized during the initial phase of rehabilitation, what would be the weekly frequency of supervised exercises? | Once a week  Twice a week  Three times per week  Four times per week  Five times per week  > Five times per week |  |
| 23- If you use resistance exercises **concentric**, in the initial phase of rehabilitation, and the patient feels pain during the exercises, would you recommend that he/she interrupt them? | Yes  No |  |
| 24- If **concentric** exercises are utilized during the initial phase of rehabilitation, how would you determine the initial load of the exercises? | 1RM test  10RM test  Manual Muscle Testing  Dynamometry assessment  According to the level of pain  According to the level of strength  Other |  |
| 25- If **concentric** exercises are utilized during the initial phase of rehabilitation, how would you determine exercise load progression? | According to the level of pain  According to the level of strength  According to the functional tests  According to the dynamometry assessment  According to the time of injury  Other |  |
| 26- If **concentric** exercises are utilized during the initial phase of rehabilitation, what would be the appropriate number of series and repetitions of the exercise? | --- |  |
| 27- If **concentric** exercises are utilized during the initial phase of rehabilitation, what would be the weekly frequency of supervised exercises? | Once a week  Twice a week  Three times per week  Four times per week  Five times per week  > Five times per week |  |
| 28- If you use resistance exercises **eccentric**, in the initial phase of rehabilitation, and the patient feels pain during the exercises, would you recommend that he/she interrupt them? | Yes  No |  |
| 29- If **eccentric** exercises are utilized during the initial phase of rehabilitation, how would you determine the initial load of the exercises? | 1RM test  10RM test  Manual Muscle Testing  Dynamometry assessment  According to the level of pain  According to the level of strength  Other |  |
| 30- If **eccentric** exercises are utilized during the initial phase of rehabilitation, how would you determine exercise load progression? | According to the level of pain  According to the level of strength  According to the functional tests  According to the dynamometry assessment  According to the time of injury  Other |  |
| 31- If **eccentric** exercises are utilized during the initial phase of rehabilitation, what would be the appropriate number of series and repetitions of the exercise? | --- |  |
| 32- If **eccentric** exercises are utilized during the initial or advanced phases of rehabilitation, what would be the weekly frequency of supervised exercises? | Once a week  Twice a week  Three times per week  Four times per week  Five times per week  > Five times per week |  |
| 33- Would you also recommend any of the exercises below during the initial phase, and if so, which one(s)? | Stretching  Shoulder mobility exercises  Scapula stabilization exercises  Exercises for the cervical and/or thoracic spine  Aerobic exercises  Proprioceptive exercises  Plyometric exercises  None of the above exercises  Other |  |
| 34- Would you recommend exercises in the advanced phase of rehabilitation to improve shoulder pain and function? | Yes  No |  |
| 35- Would you recommend resistance exercises for the rotator cuff in the advanced phase of rehabilitation to improve shoulder pain and function? | Yes  No |  |
| 36- Which resistance exercise would be most recommended for rehabilitation of shoulder tendinopathy, in the advanced phase? | Isometric  Concentric  Eccentric |  |
| 37- If you use resistance exercises **isometric**, in the advanced phase of rehabilitation, and the patient feels pain during the exercises, would you recommend that he/she interrupt them? | Yes  No |  |
| 38- If **isometric** exercises are utilized during the advanced phase of rehabilitation, how would you determine the initial load of the exercises? | Maximum isometric  Submaximum isometric  Manual Muscle Testing  Dynamometry assessment  According to the level of pain  According to the level of strength  Other |  |
| 39- If **isometric** exercises are utilized during the advanced phase of rehabilitation, how would you determine exercise load progression? | According to the level of pain  According to the level of strength  According to the functional tests  According to the dynamometry assessment  According to the time of injury  Other |  |
| 40- If **isometric** exercises are utilized during the advanced phase of rehabilitation, what would be the appropriate number of sets and duration of each isometric exercise? | --- |  |
| 41- If **isometric** exercises are utilized during the advanced phase of rehabilitation, what would be the weekly frequency of supervised exercises? | Once a week  Twice a week  Three times per week  Four times per week  Five times per week  > Five times per week |  |
| 42- If you use resistance exercises **concentric**, in the advanced phase of rehabilitation, and the patient feels pain during the exercises, would you recommend that he/she interrupt them? | Yes  No |  |
| 43- If **concentric** exercises are utilized during the advanced phase of rehabilitation, how would you determine the initial load of the exercises? | 1RM test  10RM test  Manual Muscle Testing  Dynamometry assessment  According to the level of pain  According to the level of strength  Other |  |
| 44- If **concentric** exercises are utilized during the advanced phase of rehabilitation, how would you determine exercise load progression? | According to the level of pain  According to the level of strength  According to the functional tests  According to the dynamometry assessment  According to the time of injury  Other |  |
| 45- If **concentric** exercises are utilized during the advanced phase of rehabilitation, what would be the appropriate number of series and repetitions of the exercise? | --- |  |
| 46- If **concentric** exercises are utilized during the advanced phase of rehabilitation, what would be the weekly frequency of supervised exercises? | Once a week  Twice a week  Three times per week  Four times per week  Five times per week  > Five times per week |  |
| 47- If you use resistance **eccentric** exercises, in the advanced phase of rehabilitation, and the patient feels pain during the exercises, would you recommend that he/she interrupt them? | Yes  No |  |
| 48- If **eccentric** exercises are utilized during the advanced phase of rehabilitation, how would you determine the initial load of the exercises? | 1RM test  10RM test  Manual Muscle Testing  Dynamometry assessment  According to the level of pain  According to the level of strength  Other |  |
| 49- If **eccentric** exercises are utilized during the advanced phase of rehabilitation, how would you determine exercise load progression? | According to the level of pain  According to the level of strength  According to the functional tests  According to the dynamometry assessment  According to the time of injury  Other |  |
| 50- If **eccentric** exercises are utilized during the advanced phase of rehabilitation, what would be the appropriate number of series and repetitions of the exercise? | --- |  |
| 51- If **eccentric** exercises are utilized during the advanced phase of rehabilitation, what would be the weekly frequency of supervised exercises? | Once a week  Twice a week  Three times per week  Four times per week  Five times per week  > Five times per week |  |
| 52- Would you also recommend any of the exercises below during the advanced phase, and if so, which one(s)? | Stretching  Shoulder mobility exercises  Scapular stabilization exercises  Exercises for the cervical and/or thoracic spine  Aerobic exercises  Proprioceptive exercises  Plyometric exercises  None of the above exercises  Other |  |
| 53- How often do you reassess and modify exercises for a patient with RC tendinopathy? | Would not prescribe exercises  I don’t do the progression  Weekly at least  Fortnightly  Every 3 weeks  Every month or more  Another way |  |
| 54- What is the mean number of treatment sessions required to improve pain and functional outcomes in patients diagnosed with rotator cuff tendinopathy? | < 10 sessions  10 sessions  11 – 20 sessions  21 – 30 sessions  > 30 sessions  Other |  |
| 55- What criteria do you use for discharge of patients with rotator cuff tendinopathy? | --- |  |
| 56- What adverse effects do you typically encounter using exercise for patients with rotator cuff tendinopathy? | --- |  |
| 57- In addition to exercises, what other rehabilitation techniques would you use in patients with rotator cuff tendinopathy in the initial phases of rehabilitation? | Superficial heat therapy  Deep heat therapy  Cryotherapy  Laser therapy  Pulsed ultrasound therapy  Continuous ultrasound therapy  Short waves therapy  Microwave diathermy  Shock waves therapy  Acupuncture  Dry needling  Cupping therapy  Kinesio taping  Other |  |
| 58- In addition to exercises, what other rehabilitation techniques would you use in patients with rotator cuff tendinopathy in the advanced phases of rehabilitation? | Superficial heat therapy  Deep heat therapy  Cryotherapy  Laser therapy  Pulsed ultrasound therapy  Continuous ultrasound therapy  Short waves therapy  Microwave diathermy  Shock waves therapy  Acupuncture  Dry needling  Cupping therapy  Kinesio taping  Other |  |
| 59- Would you recommend invasive interventions during the conservative treatment? | Yes  No |  |
| 60- What invasive intervention would you recommend during conservative treatment? | --- |  |
| 61- Would you recommend invasive interventions in case of failure of conservative treatment? | Yes  No |  |
| 62- What invasive intervention would you recommend in case of failure of conservative treatment? | --- |  |

**Legend:** RC = rotator cuff; 1 RM = one repetition maximum.
