# Supplementary figures and images for: Therapeutic exercises in the clinical practice of Brazilian physical therapists in the management of rotator cuff tendinopathy: An online survey
Source: PLoS One. 2024 Apr 16;19(4):e0301326. doi: 10.1371/journal.pone.0301326 (PMC11020769; doi:10.1371/journal.pone.0301326)

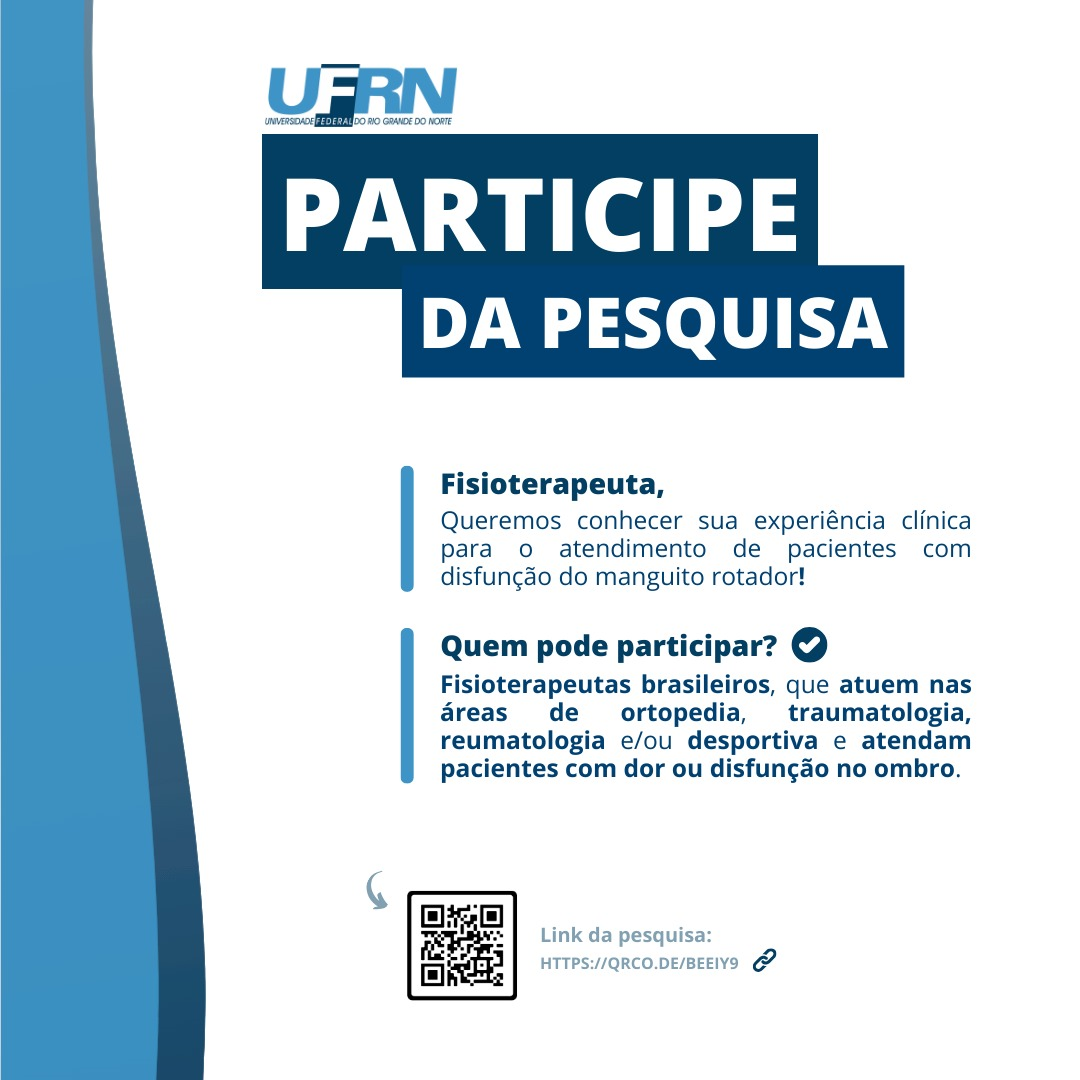

Supplement: S1 Fig — (TIF) [file pone.0301326.s004.tif]
